# Supplementary material for: A Qualitative Assessment of “Generacion Actual”: An HIV Community Mobilization Intervention Among Gay Men and Transgender Women in Lima, Peru
Source: Int J Environ Res Public Health. 2025 Nov 3;22(11):1669. doi: 10.3390/ijerph22111669 (PMC12652104; doi:10.3390/ijerph22111669)
Supplement: Supplementary file 1 [file ijerph-22-01669-s001.zip › ijerph-3872300-supplementary.pdf]

Supplementary File S1

Interview Guide for MSM/TW participating in the community intervention

Introduction

We would like to talk to you to gather information that can help improve the quality of the community building/HIV prevention program called Generación Actual that we have been conducting in Lima south. This is not an evaluation of the facilitators who are part of the program. It is very important for us to learn about the things that you liked or have worked for you in the program, what things were not useful or could be improved. Because of that, we will appreciate you being as open as possible about your experience participating in Generación Actual.

1. Can you start by telling me how you heard about the project?

2. What made you want to participate?

TRY TO GET AT LEAST 2 REASONS. Probe beyond initial responses, such as "someone asked me to, or a friend told me about it."

3. In what activities have you participated so far?

4. How does this program compared to other projects for gay men/trans you may have participated before? How is it different?

5. Let me ask you a few questions related to the project activities in which you have participated. APPLY ACCORDING TO WHAT COMPONENTS THEY HAVE PARTICIPATED:

Questions about experiential workshops

a. There is a lot of topics covered in the workshops you have attended.

Thinking of those sessions, What are three things you found useful in the sessions?

Probe for reasons

b. What about 3 things you learned in the sessions?

c. What wasn't that useful in the sessions?

d. What else would you have liked to see included in the sessions?

e. In the sessions, we talk about talking to your friends about things you have learned

in the sessions, What did you think of that?

Probe:

f. Have you been able to talk to some friends about those things?

g. If you can think back to the last time you talked to a friend in your neighborhood,

h. Can you tell me what you talked about with him?

Probe for topic they talked about, number of people involved, their comfort level, and of the friend, how the conversation got started, what person said, how friend responded.

<u>Questions about Core Group</u>

a. Tell me about the Core Group, What do you do in those meetings?

b. What do you like about the CC meetings?

c.What is the CC working on right now?

f. What have you enjoyed working with the CC?

g. What has been more difficult working with the CC?

<u>Questions about the Mobilization Activities</u>

a. What activities part of the project have you participated in?

b. Can you tell me what happened at those activities?

c. What are you getting from those activities?

d. What other kind of activities do you think would be important to organize?

<u>Questions about the Community Space</u>

a. What do you think of the community space we have for this project?

b. What kind of activities go on at the space?

c. What else do you think could be happening at the space?

d. Both gay men and trans participate in the project and go to the community center, What do you think of sharing activities/space?

<u>Changes</u>

6. If you can think of yourself before you started being part of this project and now, How has the project influenced you?

7. What changes have you observed in yourself because of being part of the project? ASK FOR EXAMPLE. Probe:

Self-reflection

Self-esteem

Changes in Communicating about sex

Changes in Condom use

Changes in how they see either gay men or trans?

8. What makes you continue participating overtime in the project?

9. What changes have you observed in other persons because of being part of the project?

10. What negative effects do you think the program has had?

Probe:

In the way your friends see you?

In the neighborhood? Something else?

11. Overall, How do you think the project can influence or impact the gay community here in Lima South?

12.Thinking of gay men/trans that you know here in Lima South, are there any groups of them who were not interested in participating in the program?

13. What changes would you like to see in the gay/trans community here?

14. How would you describe the gay/trans community here in Lima South?

14. What else do gay men/trans need here in Lima South?

15. What do you think of medical services in general here in your district?

17. How are gay-trans people treated at those medical establishments?

PROBE: How else do you think they should be treated?

DEMOGRAPHIC INFO:

If not mentioned during the interview confirm age, occupation and neighborhood of residence.

Those are all the questions we have for you. Is there anything else you would like to add that we have not talked about?
